# Amides from *Piper* as a Diuretic: Behind the Ethnopharmacological Uses of *Piper glabratum* Kunth

**DOI:** 10.1155/2014/615109

**Published:** 2014-07-01

**Authors:** Thiago Bruno Lima Prando, Tatiane da Fonseca Baciquete, Jennifer Alexandra Castanho Vieira, Jaqueline Bressan, Francielly Mourão Gasparotto, Douglas Rossi Jesus, Euclides Lara Cardozo Junior, Emerson Luiz Botelho Lourenço, Arquimedes Gasparotto Junior

**Affiliations:** ^1^Instituto de Ciências Biológicas, Médicas e da Saúde, Universidade Paranaense, P.O. Box 224, 87502-210 Umuarama, PR, Brazil; ^2^Laboratório de Farmacologia Cardiovascular, Faculdade de Ciências da Saúde, Universidade Federal da Grande Dourados, Rodovia Dourados, Itahum, km 12, P.O. Box 533, 79.804-970 Dourados, MS, Brazil

## Abstract

Several species of the genus *Piper* are known in Brazilian folk medicine as having diuretic activity. So, we propose to investigate the acute diuretic activity and the possible toxic effects of *Piper glabratum* Kunth, popularly known as false Jaborandi. Additionally, we propose to check whether there is any correlation between the biological activities of the crude extract (MEPG) and its 2-methoxy-4,5-methylenedioxy-*trans*-cinnamoyl-pyrrolidine (MMCP) in Wistar rats. The MEPG was fractioned by chromatography column and the MMCP was identified by analyses of ^1^H and ^13^C RMN spectral data and correlations. Both MEPG and MMCP were assayed for diuretic activity. The preparations obtained were orally administered in a single dose to rats. The urine excretion, pH, density, conductivity, and content of Na^+^, K^+^, Cl^−^, and HCO_3_^−^ were measured in the urine of saline-loaded animals. Additionally, acute toxicity of the extract was also evaluated. MMCP at doses of 30 mg/kg was able to increase the urine volume, pH, and HCO_3_^−^ excretion. Moreover, high dosage of MEPG showed important liver toxicity and elevated mortality when injected intraperitoneally. The results indicate that the MMCP shows important diuretic properties when administered in Wistar rats. Additionally, MEPG can induce important acute toxicity if given in high doses.

## 1. Introduction

The family Piperaceae comprises more than a thousand species distributed in tropical and subtropical regions of both hemispheres, being represented by herbaceous plants, shrubs, and less often trees. It is distributed in eight genera with the genus* Piper*, with about 2000 species, being the most representative [1].

The* Piper* species are popularly known in Brazil as pepper, pariparoba caapeba, and false jaborandi, among others [2]. Chemically these species have been widely investigated as promising sources of secondary metabolites with antiparasitic, anticonvulsant, anti-inflammatory, and diuretic activities [3, 4]. Phytochemical investigations have led to the identification of typical classes of compounds such as amides [5, 6], terpenes, benzoic acid derivatives, carotenoids, and hydroquinones as well as lignans, neolignans, and some alkaloids [7].

Despite the importance of the genus* Piper*, little information is found on* Piper *species, native of southern Brazil. Among others, studies on* Piper glabratum* Kunth are very limited, considering the chemical and pharmacological possibilities of this species. Recently, Flores et al. [8] showed that benzoic acid derivatives from* P. glabratum* have antiparasitic activity against* Leishmania* spp. and* Trypanosoma cruzi*. Nevertheless, the possible diuretic effects of this species still need to be investigated. So, considering their popular use, we propose to evaluate the acute diuretic activity and the possible toxic effects of methanolic extract from* P. glabratum*. Furthermore, we propose to check whether there is any correlation between the biological activities of the crude extract and its 2-methoxy-4,5-methylenedioxy-*trans*-cinamoyl-pyrrolidine (MMCP) in Wistar rats.

## 2. Materials and Methods

### 2.1. Drugs and Chemicals

Hydrochlorothiazide (HCTZ) was obtained from Sigma-Aldrich Chemical Co. (St. Louis, MO, USA). All other reagents were of analytical grade.

### 2.2. Phytochemical Study

#### 2.2.1. General

Silica gel (60–230, 230–400 mesh) (Merck) was used for chromatography column (CC) and silica gel 60F_254_ (Merck) for preparative thin layer chromatography (TLC). The solvents *n*-hexane, dichloromethane, ethyl acetate, and methanol (Vetec) were used for extraction and partition. ^1^H NMR (300 MHz) and ^13^C NMR (100 MHz) were obtained in CDCl_3_ (BRUKER, mod. AMX-300). Several one- and two-dimensional NMR experiments were used to establish through-bond connectivity. Heteronuclear multiple quantum correlation (^13^C HMQC) spectroscopy was used to identify bonded proton-carbon pairs. Heteronuclear multiple bond correlation (^13^C HMBC) spectroscopy was employed to delineate long-range proton-carbon bonded interactions.

#### 2.2.2. Plant Material

*P. glabratum* roots were collected from the* “Refúgio Biológico Bela Vista”* (Foz do Iguaçu/PR, Brazil) at 164 m above sea level (S25°26′48′′-W54°33′06′′). The plant was identified by Dra. Sonia Regina Hefler (Pontifical Catholic University of Paraná, Brazil). Voucher specimens were deposited at the herbarium of this university under number 19.434.

#### 2.2.3. Preparation of the Methanolic Extract of* P. glabratum* (MEPG) and Isolation of Pyrrolidine Amide

*P. glabratum *roots were dried in a forced draft oven (45°C, 48 h). In sequence, the roots were crushed and pulverized. The MEPG was prepared by maceration (1 : 5 w/v) from methanol/water (70 : 30 v/v) at room temperature for seven days. The preparations were filtered, concentrated in rotatory evaporator, lyophilized, and stored at −20°C until the pharmacological experiments.

For isolation of pyrrolidine amide, a sample of MEPG was subjected to CC with *n*-hexane, dichloromethane, and methanol, respectively. The dichloromethane fraction was subjected to silica gel CC, eluting initially with *n*-hexane and gradually enriching with dichloromethane, to obtain 22 fractions. Fraction A-33/37 (1.551 g, *n*-hexane-dichloromethane, 3 : 7) was applied to a silica gel column, eluting with *n*-hexane and gradually increasing the polarity with EtOAc, to obtain 21 fractions. Fraction A-23/63 (333 mg, *n*-hexane-EtOAc, 4 : 6) was subjected to TLC (*n*-hexane-EtOAc, 4 : 6) to afford white amorphous solid (**P-1**, 56.4 mg).** P-1** showed signals related to methylene protons of pyrrolidine rings (Table 1), confirmed by nuclear magnetic resonance (^1^H NMR, ^13^C NMR, HMQC, and HMBC) as a 2-methoxy-4,5-methylenedioxy-*trans*-cinnamoyl-pyrrolidine (MMCP).

### 2.3. Pharmacological Studies

#### 2.3.1. Animals

We used male and female Wistar rats (3-4 months old) from the colony of the Universidade Paranaense. The animals were maintained with a constant 12 h light/dark cycle and controlled temperature (22 ± 2°C). Standard pellet food (Nuvital, Curitiba/PR, Brazil) and water were available* ad libitum*. All experimental procedures were previously approved by the Institutional Ethics Committee of the Universidade Paranaense (authorization 20763-2011).

#### 2.3.2. Assessment of Acute Diuretic Activity (Single-Dose Study)

The diuretic activity was determined according to the method previously described [9]. Female Wistar rats were separated in different groups (*n* = 6) and fasted overnight with free access to water. Before the treatments, all animals received physiological saline (0.9% NaCl) in an oral dose of 5 mL/100 g to impose a uniform water and salt load. Then, the first group received vehicle (deionized water) orally and it was used as control. Other groups of rats received, by oral route, MEPG (30, 100, and 300 mg/kg), MMCP (3, 10, and 30 mg/kg), or HCTZ (hydrochlorothiazide, 10 mg/kg). The urine was collected in a graduated cylinder and its volume was recorded for 8 h (expressed as mL/100 g). At the end of the experiments serum samples were obtained by decapitation for measurement of Na^+^ and K^+^. Electrolyte concentrations were quantified by flame photometry (Na^+^ and K^+^) or titration (Cl^−^ and HCO_3_^−^). pH and conductivity were directly determined on fresh urine samples using a Q400MT pH-meter and a Q795 M2 conductivity meter (Quimis Instruments, Brazil), respectively. Density estimation was made by weighing with a Mettler AE163 (±0.1 mg) analytical balance.

#### 2.3.3. Evaluation of Acute Toxicity

Female and male rats (*n* = 6) were orally (100, 300, 1000, and 3000 mg/kg) and intraperitoneally (100, 300, 1000, and 3000 mg/kg) submitted to a single dose of MEPG. After treatment, the animals were observed for the first hour, followed by every hour up to 6 h, and subsequently daily for 14 days. The body weight, food and water consumption, and the number of deaths (expressed as LD50) were recorded daily throughout the trial period. At the end of experimental period (14 days) the animals were euthanized by decapitation and samples of serum were obtained for measurement of urea, creatinine, alanine transaminase (ALT), and aspartate transaminase (AST) by enzymatic method (Cobas Mira, Roche, Indianapolis, USA). The liver of all animals was removed and weighed. The weight of liver was multiplied by 100 and divided by the weight of the animal before euthanasia to obtain the relative organs weight (%) [10].

### 2.4. Statistical Analysis

The data obtained were processed through variance analysis (ANOVA), followed by Bonferroni's test, in those cases in which samples were normal and homoscedastic. For the data without homoscedastic samples and normal distribution, the nonparametric Kruskal-Wallis test was used, followed by the Mann-Whitney test. The acute oral LD50 of the extract was calculated by the use of software for probit analysis. The significance level was *P* < 0.05.

## 3. Results

### 3.1. Chemical Identification

The* P. glabratum* Kunth roots were extracted with methanol/water and successive chromatographic columns resolution over silica gel. A solid substance (**P-1**) was obtained from dichloromethane fraction. 300 MHz ^1^H NMR, 100 MHz ^13^C NMR, HMQC, and HMBC (Table 1) have indicated 2-methoxy-4,5-methylenedioxy-*trans*-cinnamoyl-pyrrolidine (MMCP). The spectrum analysis indicated the presence of methylene protons, *δ*_H_ 1.85 (2H, *m*, *J* = 16.6), 1.96 (2H, *m*, *J* = 15.3), 3.55 (2H, *m*, *J* = 32.2), and 3.57 (2H, *m*, *J* = 32.2); ^13^C NMR signals *δ*_C_ 46.50, 45.93, 26.15, and 24.37 confirmed the presence of a pyrrolidine moiety. The signals *δ*_H_ 6.57 (1H, *d*, *J* = 16) and 7.90 (1H, *d*, *J* = 16) and *δ*_C_ 165.41, 136.54, and 117.06 show the *α*, *β*-unsaturated carbonyl, with* trans* conformation. The ^13^C NMR chemical shifts, *δ*_C_ 94.86, 106.67, 117.09, 141.51, 149.71, and 154.65, and the ^1^H NMR signals *δ*_H_ 6.49 (1H, *s*, *J* = 16.08) and 6.96 (1H, *s*, *J* = 16.25) demonstrate the presence of the substituted aromatic system. The signals *δ*H 5.91 (2H, *s*, *J* = 35.98) and 3.78 (3H, *s*, *J* = 22.76) added to ^13^C NMR signals *δ*_C_ 56.57 and 101.57 confirm the methoxyl and methylenedioxy substitutions groups. The signal assignments were confirmed by correlations observed in the HMQC and HMBC spectra.

### 3.2. Acute Diuretic Activity

The effects of acute treatment with MEPG (30, 100, and 300 mg/kg), MMCP (3, 10, and 30 mg/kg), and hydrochlorothiazide on urine volume and electrolyte (Na^+^, K^+^, Cl^−^, and HCO_3_^−^) excretion are presented in Table 2. Urine volume and electrolytes excretion were not significantly altered by the administration of MEPG. On the other hand, MMCP administration (30 mg/kg) was able to induce a substantial increase in urine volume, urinary HCO_3_^−^, and urine pH. All other evaluated parameters did not show significant differences when compared to the control group. The levels of Na^+^ and K^+^ in plasma, measured at the end of the experiment (8 h), were not affected by any treatment.

### 3.3. Toxicological Findings

All parameters related to acute toxicity are presented in Table 3. In behavioral assessment after treatment, for up to 6 hours after administration, we can observe that all animals treated with doses of 1000 and 3000 mg/kg (intraperitoneally) and 3000 mg/kg (per oral route) had expressive sedation, characterized by decreased spontaneous movement or induced by touch. Besides, we observed that animals treated with MEPG at 3000 mg/kg (intraperitoneally) showed a significant decrease in body weight and consumption of water and food and an expressive increase in liver transaminases (ALT and AST). The LD50 per intraperitoneal route (3000 mg/kg) was found to be 2426.216 mg/kg in female rats, while in male rats it was over 3000 mg/kg. In all animals treated by oral route any occurrence of death was not observed, featuring LD50 as above 3000 mg/kg. Serum urea and creatinine were not affected by any of the treatments.

## 4. Discussion

*Piper,* the pepper plants or pepper vines, is an economically and ecologically important genus in the Piperaceae family. The genus contains species suitable for studying natural history, molecular biology, natural products chemistry, and evolutionary biology [1]. Despite its importance, few studies have been conducted on native species from southern Brazil, especially as to its use as a diuretic. So, this was the first study that showed that* P. glabratum *extract (MEPG) has no diuretic activity when administered orally. Additionally, we showed that high doses of this extract can cause liver toxicity and high mortality when administered by intraperitoneal route. Nevertheless, we isolated and identified a pyrrolidine amide (MMCP) which induced an important increase in urine volume and bicarbonate excretion and elevated the urine pH.

*Piper* amides are abundant in this genus and have great ecological and economic importance, mainly as cytotoxic, insecticidal, fungicidal, or other economically important activities. The majority of these compounds are composed of an acid such as cinnamic acid forming an amide where the nitrogen is in a five- or a six-membered ring or on an isobutyl chain [1]. The MMCP was previously isolated from* P. amalago* [5] and* P. peepuloides* [6] and unprecedented in* P. glabratum*.

Since the 1940s different amides or derivatives have been investigated as potential diuretic agents [11], and in the last fifty years different amide derivatives, especially sulfonamides, have been used as diuretic. Drugs of great clinical relevance as furosemide, hydrochlorothiazide, and acetazolamide are worth mentioning. In most cases, sulfonamides have the ability to excrete sodium chloride and water. On the other hand, some molecules known as carbonic anhydrase inhibitors (CAI) also excrete large quantities of bicarbonate [12]. Although the purpose of comparing the MMCP with the hydrochlorothiazide had not shown similar results, we observed that the diuretic response induced by MMCP was closest to the effects of acetazolamide, a CAI. In a recent study performed by Gasparotto Jr. et al. [13] it was shown that 10 mg/kg of acetazolamide is capable of raising the pH and increasing the excretion of bicarbonate, sodium, and potassium in Wistar rats. Thus, it may be possible that lower capacities of MMCP to excrete sodium and potassium reflect the reduced ability of this amide in interacting with the active center of CA, or this effect may involve other mechanisms not yet elucidated. Furthermore, the fact that MEPG presents small amounts of MMCP may, at least partly, explain the absence of diuretic activity of MEPG.

Despite promising data concerning diuretic properties of the amides of this species, we evidenced important toxic effects of MEPG. After intraperitoneal administration, MEPG induced an important reduction in body weight and water and food intake. Moreover, important alterations in liver function tests (elevation of liver transaminases) were observed and might indicate liver hepatocellular injury due to increased membrane permeability or cell necrosis [14, 15]. Another parameter that caught our attention was the mortality rate. In our study the LD50 value of the MEPG on male rats was superior to that found for the females. This difference may involve factors which account for gender-related pharmacokinetic differences. Furthermore, the fact that orally the MEPG presents an LD50 above 3000 mg/kg may be due to difference in route of entry, which allows the drug to undergo presystemic metabolism before reaching the target tissue [16].

## 5. Conclusion

In conclusion, the present study does not support the traditional usage of* P. glabratum* in the Brazilian folk medicine as a diuretic agent. Additionally, we have confirmed that a possible diuretic activity may be associated with the presence of the MMCP, which showed a saluretic/diuretic effect. Furthermore, our results provide evidence for the toxicity profile of the MEPG at high doses and therefore it should be ingested with caution. Further studies should be conducted to assess the possible mechanisms by which the* Piper *amides exert their diuretic effects and the role of these agents in toxic effects of MEPG.

## Figures and Tables

**Table 1:** ^
13^C NMR (100 MHz, CDCl_3_), ^1^H NMR (300 MHz, CDCl_3_), ^1^H, ^13^C–HMQC, and 1H, ^13^C–HMBC data of **P-1** compound.

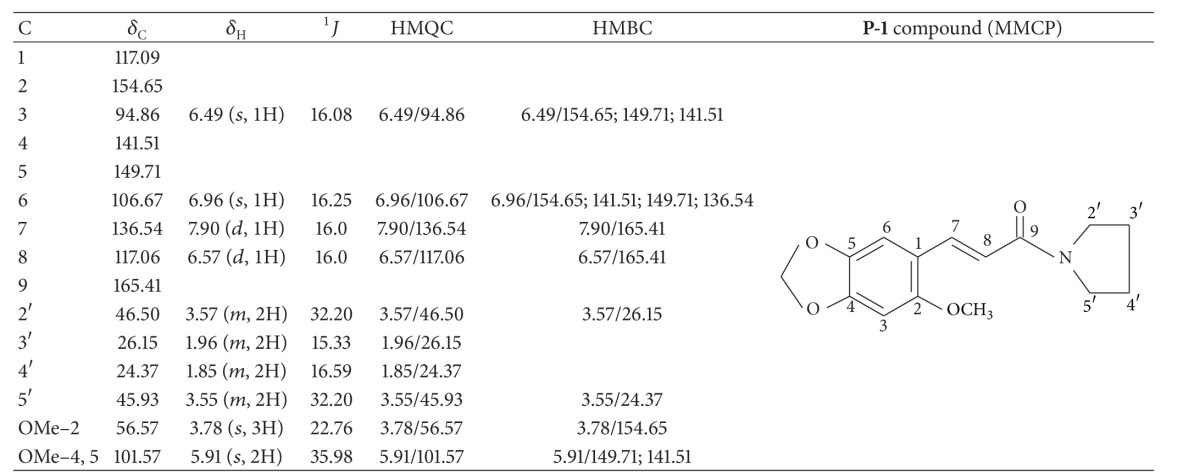

Chemical shifts *δ* in ppm relative to TMS, *J* in Hz.

**Table 2:** Effect of acute oral administration of methanolic extract from *Piper  glabratum* (MEPG) and its pyrrolidine amide (MMCP) on urinary volume, electrolyte excretion, pH, and conductivity.

Group	Urine (mL/8 h/100 g)	Na^+^ (mmol/L)	K^+^ (mmol/L)	Cl^−^ (mmol/L)	HCO_3_^−^ (mmol/L)	pH	Conductivity (mS/cm)
Control	2.15 ± 0.3	106.0 ± 4.1	37.0 ± 6.4	145.0 ± 12.5	225.0 ± 19.5	6.0 ± 0.4	14.3 ± 0.5
HCTZ (10 mg/kg)	3.96 ± 0.5^a^	140.3 ± 8.6^a^	55.5 ± 4.3^a^	160.0 ± 11.1	257.0 ± 18.1	5.9 ± 0.2	16.3 ± 0.7^a^
MEPG (30 mg/kg)	2.52 ± 0.2	114.5 ± 6.1	45.7 ± 2.7	150.0 ± 26.4	230.0 ± 25.0	6.0 ± 0.2	15.0 ± 1.1
MEPG (100 mg/kg)	2.24 ± 0.4	99.7 ± 9.9	36.2 ± 9.7	130.0 ± 28.8	245.0 ± 27.5	6.0 ± 0.2	13.7 ± 2.2
MEPG (300 mg/kg)	2.28 ± 0.4	100.0 ± 8.7	36.5 ± 6.1	125.0 ± 10.5	250.0 ± 11.5	5.7 ± 0.3	14.8 ± 1.6
MMCP (3 mg/kg)	2.51 ± 0.2	99.5 ± 6.2	34.0 ± 4.8	155.0 ± 12.5	280.0 ± 18.9	5.8 ± 0.2	13.5 ± 1.2
MMCP (10 mg/kg)	2.60 ± 0.3	94.0 ± 9.0	48.6 ± 6.4	140.0 ± 23.0	315.0 ± 42.7	6.7 ± 0.3	15.0 ± 1.3
MMCP (30 mg/kg)	3.30 ± 0.33^a^	120.7 ± 8.3	41.8 ± 6.6	125.0 ± 15.0	380.0 ± 11.5^a^	8.4 ± 0.3^a^	15.9 ± 0.9

Values are expressed as mean ± SEM of six rats in each group in comparison to the control using one-way ANOVA followed by Bonferroni's test (^a^ *P* < 0.05). HCTZ: hydrochlorothiazide; MMCP: 2-methoxy-4, 5-methylenedioxy-*trans*-cinnamoyl-pyrrolidine.

**Table 3:** Acute toxicity variables after treatment with methanolic extract from *Piper glabratum* (MEPG) in male and female Wistar rats.

Parameters	Experimental groups			
	Control	MEPG (1 g/kg; i.p.)	MEPG (3 g/kg; i.p.)	MEPG (3 g/kg; p.o.)
Body weights (g), males	330.2 ± 3.7	322.7 ± 4.0	285.1 ± 2.7^a^	316.1 ± 4.6
Water intake (g), males	233.6 ± 7.6	240.4 ± 9.2	175.7 ± 9.9^a^	244.6 ± 8.4
Food intake (g), males	151.6 ± 5.6	122.7 ± 7.9	114.9 ± 6.7^a^	145.1 ± 4.5
Body weights (g), females	274.0 ± 1.3	268.6 ± 2.4	243.2 ± 3.1^a^	277.5 ± 1.3
Water intake (g), females	199.7 ± 11.3	142.3 ± 7.6	134.1 ± 6.5^a^	202.0 ± 7.1
Food intake (g), females	122.1 ± 4.7	118.4 ± 8.7	91.5 ± 5.6^a^	117.1 ± 3.8
Relative liver weights				
Liver (%), males	4.13 ± 0.19	4.33 ± 0.32	3.96 ± 0.07	3.91 ± 0.09
Liver (%), females	2.79 ± 0.24	3.13 ± 0.24	2.46 ± 0.04	2.72 ± 0.18
Liver function tests				
AST (mg/dL), males	111.4 ± 8.3	163.8 ± 11.9	289.5 ± 11.3^a^	159.0 ± 29.9
ALT (mg/dL), males	63.0 ± 5.1	81.7 ± 3.3	216.8 ± 31.7^a^	90.80 ± 9.7
AST (mg/dL), females	107.6 ± 8.1	100.0 ± 11.9	181.5 ± 14.0^a^	101.2 ± 6.0
ALT (mg/dL), females	46.0 ± 4.0	40.2 ± 5.6	122.0 ± 21.6^a^	51.4 ± 1.8

Values are expressed as mean ± SEM of six rats in each group in comparison to the control using one-way ANOVA followed by Bonferroni's test or Kruskal-Wallis test followed by Mann-Whitney test (relative liver weights) (^a^ *P* < 0.05). AST: aspartate transaminase; ALT: alanine transaminase; i.p.: intraperitoneally; p.o.: per oral route.
